# A key antisense sRNA modulates the oxidative stress response and virulence in *Xanthomonas oryzae* pv. *oryzicola*

**DOI:** 10.1371/journal.ppat.1009762

**Published:** 2021-07-23

**Authors:** Yan Wu, Sai Wang, Wenhan Nie, Peihong Wang, Luoyi Fu, Iftikhar Ahmad, Bo Zhu, Gongyou Chen

**Affiliations:** 1 Shanghai Yangtze River Delta Eco-Environmental Change and Management Observation and Research Station, Ministry of Science and Technology, Ministry of Education, Shanghai Urban Forest Ecosystem Research Station, National Forestry and Grassland Administration, Shanghai Cooperative Innovation Center for Modern Seed Industry, School of Agriculture and Biology, Shanghai Jiao Tong University, Shanghai, China; 2 Department of Environmental Sciences, COMSATS University Islamabad, Vehari Campus, Vehari, Pakistan; University of Florida Institute of Food and Agricultural Sciences, UNITED STATES

## Abstract

Pathogens integrate multiple environmental signals to navigate the host and control the expression of virulence genes. In this process, small regulatory noncoding RNAs (sRNAs) may function in gene expression as post-transcriptional regulators. In this study, the sRNA Xonc3711 functioned in the response of the rice pathogen, *Xanthomonas oryzae* pv. *oryzicola* (*Xoc*), to oxidative stress. Xonc3711 repressed production of the DNA-binding protein Xoc_3982 by binding to the *xoc_3982* mRNA within the coding region. Mutational analysis showed that regulation required an antisense interaction between Xonc3711 and *xoc_3982* mRNA, and RNase E was needed for degradation of the *xoc_3982* transcript. Deletion of Xonc3711 resulted in a lower tolerance to oxidative stress due to the repression of flagella-associated genes and reduced biofilm formation. Furthermore, ChIP-seq and electrophoretic mobility shift assays showed that Xoc_3982 repressed the transcription of effector *xopC2*, which contributes to virulence in *Xoc* BLS256. This study describes how sRNA Xonc3711 modulates multiple traits in *Xoc* via signals perceived from the external environment.

## Author summary

Small, stable RNA species perform diverse functions in both prokaryotes and eukaryotes. In this study, the sRNA Xonc3711 decreased the production of DNA-binding protein Xoc_3982 in the bacterium *Xanthomonas oryzae* pv. *oryzicola* (*Xoc*) by base pairing with the *xoc_3982* transcript. When Xonc3711 was mutated, *Xoc* was impaired in its ability to form flagella and produce biofilms, which reduced *Xoc* tolerance to oxidative stress. We also discovered that the DNA-binding protein Xoc_3982 represses the expression of *xopC2*, which encodes an effector protein, and reduces its expression. Our results show that Xonc3711 modulates and integrates multiple systems in *Xoc* to protect cells from oxidative damage.

## Introduction

Bacterial pathogens can adapt to stressful conditions by altering the activity and number of transcriptional regulators [1–3]. For example, regulatory proteins may be modulated at the transcriptional level or subjected to post-translational modifications such as phosphorylation and glycosylation [4, 5]. In addition, global regulators of gene expression can be modulated by small regulatory RNA (sRNA) molecules that target mRNA at the post-transcriptional level via base pairing; this ultimately controls gene expression of the target and can impact virulence [6]. In prokaryotes, the sRNAs that base pair with target mRNAs can be further assigned into two subgroups: cis- and trans-encoded sRNAs [7]. The cis-encoded RNAs are transcribed from the complementary strands of their target; this group is often encoded by phages, plasmids and transposons and includes sRNAs that are classified as riboswitches [7]. The trans-encoded sRNAs have been extensively studied in prokaryotes; these sRNAs are transcribed from genomic loci that are physically separate from their target genes. The trans-encoded sRNAs generally mediate translation or stability of target mRNAs by partial or discontinuous base pairing [7].

sRNAs can regulate target genes positively or negatively. For example, positive regulation of the target gene may occur when sRNAs base pair with the target mRNA, which can unmask the ribosome binding site (RBS) in the target and promote its translation. Alternatively, sRNAs can negatively regulate their targets by inhibiting translation and/or stimulating degradation via ribonuclease RNase E [8]. The interaction of sRNA with target mRNA generally requires the RNA chaperone Hfq, which binds sRNAs, facilitates sRNA-mRNA base pairing, and directly binds and regulates translation of certain mRNAs [9].

One of the earliest cellular reactions to pathogen invasion and recognition is the generation of reactive oxygen species (ROS) by the host; this includes the superoxide anion (O_2_^-^) and its dismutation product, hydrogen peroxide (H_2_O_2_) [10]. sRNAs can regulate pathogen metabolism by targeting a wide range of virulence factors and stress-response proteins to evade immune defenses and colonize their host. Bacterial sRNAs play major roles in stress tolerance both inside and outside the host cell and promote survival during suboptimal conditions [11, 12]. For example, the sRNA RsaC modulates the oxidative stress response of *Staphylococcus aureus* during manganese starvation by repressing the translation of the Mn-containing enzyme SodA [13]. The sRNA DicF promotes the expression of genes in the type III secretion system (T3SS) in *Escherichia coli* O157:H7 under oxygen-limited conditions [14]. The sRNA OxyS integrates the oxidative stress response with other cellular responses to help protect *E*. *coli* from oxidative damage [15].

The gram-negative plant pathogen, *Xanthomonas oryzae* pv. *oryzicola* (*Xoc*), causes bacterial leaf streak in rice and is an important organism for studying plant-microbe interactions. Many regulatory genes have been characterized in *Xoc*, especially genes mediating pathogenicity and recognition of host plants. However, relatively few studies have documented the importance of sRNAs and sRNA-mediated regulation in *Xanthomonas* spp. In *X*. *campestris* pv. *campestris*, transcription of sRNA-Xcc1 was shown to be modulated by the T3SS regulators, HrpG and HrpX, indicating that sRNA-Xcc1 may have a regulatory role in virulence [16]. In the related pathogen *X*. *campestris* pv. *vesicatoria*, sRNA sX13 showed potential regulatory roles in motility and transcriptional regulation of virulence genes [17]. In *X*. *oryzae* pv. *oryzae* (*Xoo*), which is closely related to *Xoc*, a recent study identified sRNAs trans217 and trans3287 as virulence-associated sRNAs that are required for pathogenicity in susceptible rice plants and for the elicitation of the hypersensitive response in nonhost plants. The authors suggested that these sRNAs directly regulate the T3SS in *Xoo* [18]. In a prior study [19], eight sRNAs were functionally characterized in *Xoo*; among these, sRNA-Xoo1 was of special interest because it was conserved in other *Xanthomonas* spp., and its expression was Hfq-dependent. Analysis of a sRNA-Xoo1 mutant revealed down-regulated levels of superoxide dismutase, which suggests a potential regulatory role in oxidative stress.

Our lab is interested in the role of post-transcriptional RNA regulation and editing in *Xoc*, especially with respect to pathogenicity, motility, biofilm formation and adaptation to oxidative stress [20]. This study focuses on sRNA Xonc3711, which is the *Xoc* homolog of sRNA-Xoo1; as mentioned above, sRNA-Xoo1 was responsive to oxidative stress [19]. In the current study, we show that Xonc3711 plays an extensive role in modulating *Xoc* transcription during oxidative stress and biosynthesis of flagella. sRNA Xonc3711 interacts with the mRNA of *xoc_3982*, which encodes a DNA-binding protein. Furthermore, Xoc_3982 binds to the promoter region of the T3SS effector *xopC2* to modulate the virulence of *Xoc* BLS256. Our results confirm a role for sRNA Xonc3711 in regulating multiple systems in *Xoc*.

## Results

### sRNA Xonc3711 targets *xoc_3982* mRNA

Liang et al. previously reported that the small RNA designated sRNA-Xoo1 was conserved in *Xoc* strain BLS256 [19]. The homologue of sRNA-Xoo1 in *Xoc* BL526 maps adjacent to *Xoc_3711*, which encodes a hypothetical protein (S1B Fig). Due to the proximity of the sRNA to Xoc_3711, it was named Xonc3711, with the ‘nc’ indicating that it is non-coding RNA. In preliminary experiments, the expression of *xonc3711* was significantly upregulated in the presence of 0.1 mM H_2_0_2_ (S1C Fig), indicating a potential role in oxidative stress. The secondary structure of Xonc3711 was predicted using software available at https://sfold.wadsworth.org (S1A Fig).

**Fig 1:**
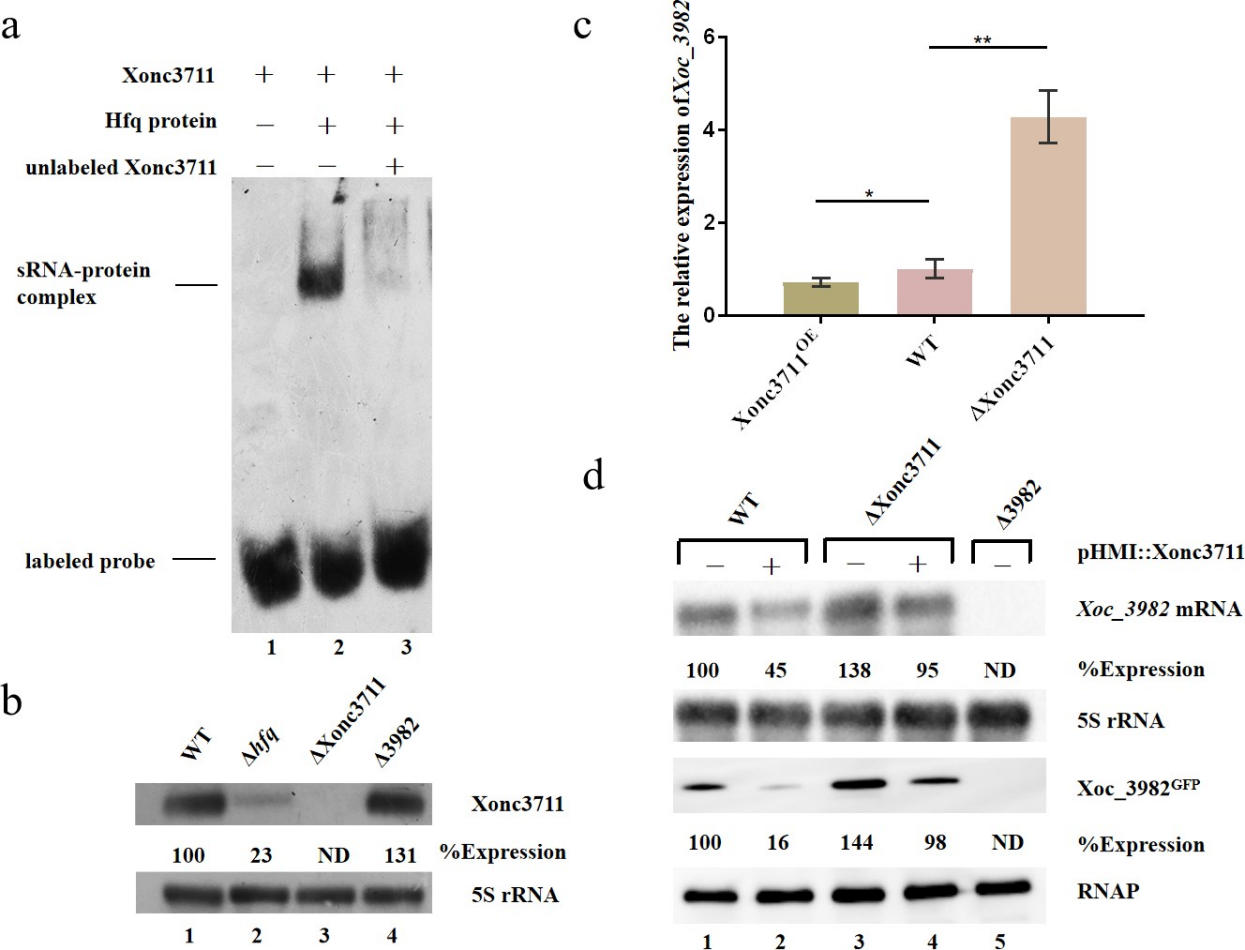
sRNA Xonc3711 binds Hfq and targets the *Xoc_3982* mRNA in *Xoc* BLS256. (a) Interaction between Xonc3711 and Hfq protein by EMSA. Lane 1 contains 3’-biotinylated Xonc3711; lane 2, contains biotinylated Xonc3711 and Hfq protein; and lane 3, consists of biotinylated Xonc3711, Hfq protein, and unlabeled Xonc3711. (b) Northern blot analysis of Xonc3711 expression in wild-type *Xoc* BLS256, ΔHfq, ΔXonc3711 and ΔXoc_3982 strains grown to OD_600_ = 1.0 in NB. 5S rRNA was used as a loading control, and Image J was used to calculate expression levels. The intensity of the band in the first lane (WT) was normalized to a value of 100. (c) qRT-PCR analysis of *xoc_3982* expression in *Xoc* BLS256 overexpressing Xonc3711 (Xonc3711^OE^), wild-type BLS256 (WT) and the ΔXonc3711 mutant. Expression levels of target genes were calculated relative to *rpoD* using the ΔΔCT method, where CT is the threshold cycle. Four independent biological replicates were carried out in this study (Wilcoxon-Mann-Whitney test). (d) Northern (upper two panels) and western (lower two panels) blot analysis of *Xoc_3982* mRNA and protein levels, respectively, in BLS256 (WT), ΔXonc3711, and Δ3982 strains. In lanes labeled with (+), Xonc3711 was overexpressed from the pHM1::Xonc3711 construct. Expression of 5S rRNA and levels of RNAP were used as loading controls for northern and western blots, respectively. Values above each band represent band intensity and were calculated using Image J software. Band intensity in the first lane was normalized as 100. ND, not detected.

Previous results with sRNA-Xoo1 indicated that expression or stability of this small RNA was dependent on the RNA chaperone, Hfq [19]. Thus, we used electrophoretic mobility shift assays (EMSA) to evaluate whether Xonc3711 and Hfq interacted *in vitro*. EMSA clearly indicated a strong interaction between biotinylated Xonc3711 and Hfq (Fig 1A). To evaluate whether Hfq impacted Xonc3711 transcription, expression was compared in the wild-type BL526 (WT), a *hfq* deletion mutant (*Δhfq*), and a Xonc3711 deletion mutant (ΔXonc3711) (Fig 1B). There was a substantial decrease in Xonc3711 transcription in the *Δhfq* mutant, indicating that Hfq has a role in the expression of the sRNA Xonc3711.

*In silico* searches were performed to identify Xonc3711 targets with the CopraRNA algorithm using Xonc3711 sequence as the query and the *Xoc* BLS256 genome as the target [21]. Using this approach, Xonc3711 was predicted to target Xoc_3982, a putative DNA-binding protein. To evaluate whether Xonc3711 and Xoc_3982 interact, the expression of *xoc_3982* was assessed in *Xoc* BL256 (WT), a strain overexpressing Xonc3711 (Xonc3711^OE^), and the ΔXonc3711 mutant (Fig 1C). Expression of *xoc_3982* in the Xonc3711^OE^ strain was much lower than expression in the ΔXonc3711 mutant, which suggests that Xonc3711 may promote *xoc_3982* mRNA degradation. Expression of *xoc_3982* was then compared in WT and ΔXonc3711 mutant strains with and without overexpression of Xonc3711; a deletion mutant in *xoc_3982* was included as a control. Western blot analysis showed that Xoc_3982 protein levels were elevated in ΔXonc3711, and overexpression of Xonc3711 caused a reduction in Xoc_3982 protein levels (Fig 1D). Northern blot results correlated with the western analyses and showed elevated expression of *xoc_3982* transcripts in the ΔXonc3711 mutant (Fig 1D).

### Post-transcriptional regulation of *xoc_3982*

**Fig 2:**
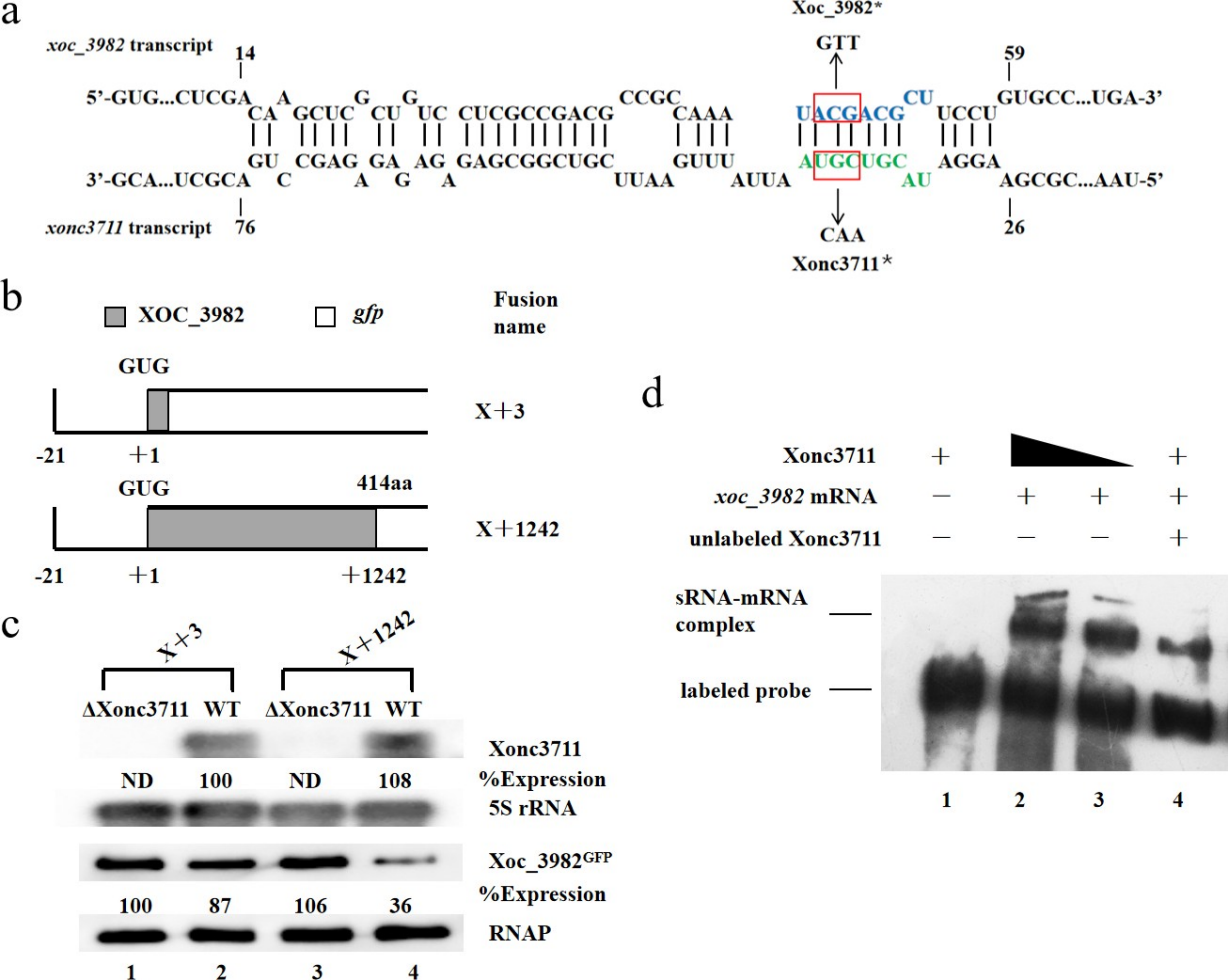
Analysis of the Xonc3711-*xoc_3982* RNA interaction. (a) Nucleotide sequence of BLS256 *xoc_3982* mRNA from its initiation codon (+3) to +1245 in the CDS, with those of the predicted export signal sequence in blue font. Blue panel: The location of point mutations in Xoc_3982*; Green panel: The location of point mutations in Xonc3711*. (b) Schematic showing *Xoc_3982*::*gfp* translational fusions. Two fusions were constructed that varied in the number of nucleotides in the coding sequence. Constructs X+3 and X+1242 contained three and 1242 nucleotides of the *Xoc_3982* coding sequence fused to GFP, respectively. (c) Northern blot showing Xonc3711 expression levels in the presence of reporter constructs X+3 and X+1242 (upper two panels). The lower two panels show Xoc_3982^GFP^ proteins detected by western blotting. GFP fusion proteins were detected using antibodies directed against GFP (1:2000, mouse anti-GFP; Roche). (d) Interaction of Xonc3711 sRNA and full-length *xoc_3982* mRNA in electrophoretic mobility shift assays. Lane 1, biotinylated Xonc3711; lane 2, biotinylated Xonc3711 and 40 μM *xoc_3982* mRNA; lane 3, biotinylated Xonc3711 and 20 μM *xoc_3982* mRNA; and lane 4, biotinylated Xonc3711, *xoc_3982* mRNA, and unlabeled Xonc3711.

Hfq-dependent sRNAs activate or repress mRNA targets by several methods [6]. One regulatory mechanism includes base-pairing between the sRNA with the coding sequence (CDS) of the target mRNA, which inhibits translation [22]. We predicted that nucleotides 14–59 of the *xoc_3982* mRNA target sequence would be complementary with the Xonc3711 seed region (Fig 2A). To test this hypothesis, the start codon of *xoc_3982* (region +3 relative to the GUG; Fig 2B) was translationally fused to green fluorescent protein (GFP), resulting in construct X+3 (Fig 2B). Xonc3711 failed to regulate the X+3 reporter since both RNA and Xoc_3982 protein levels remained unchanged with this fusion (Fig 2C, lanes 1 and 2). When Xonc3711 was paired with the X+1242 reporter fusion, RNA levels remained unchanged, but Xoc3982 protein levels were repressed relative to the controls (lanes 3 and 4); this result supported our prediction that Xonc3711 targeted a region within the *xoc_3982* CDS. Furthermore, biotinylated Xonc3711 interacted with full-length *xoc_3982* mRNA in gel shift assays (Fig 2D, lanes 2 and 3). These findings indicate that *xoc_3982* is the target of sRNA Xonc3711, which regulates *xoc_3982* mRNA after transcription by base pairing with the *xoc_3982* CDS.

### Short CDS pairing is essential for *xoc_3982* repression

**Fig 3:**
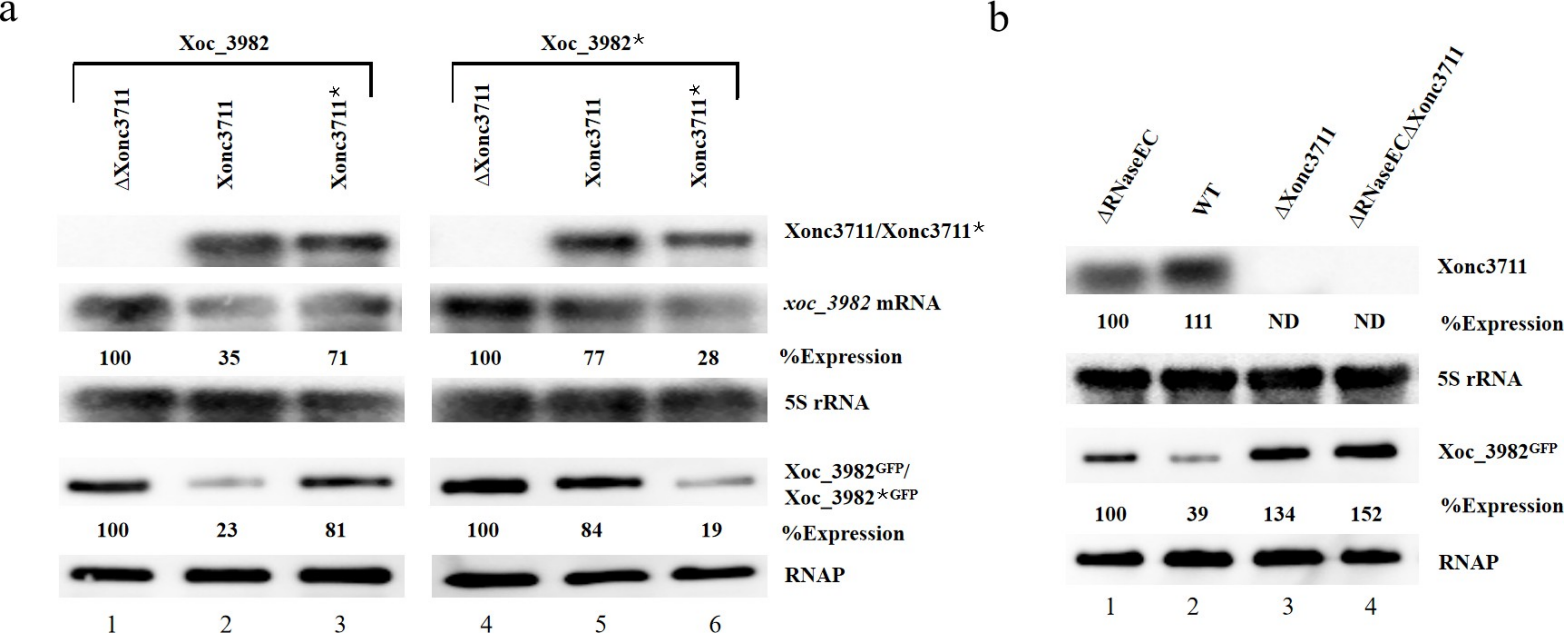
Validation of the Xonc3711-*xoc_3982* RNA interaction *in vivo* and requirement of RNase E for degradation. (a) Compensatory point mutations validate the Xonc3711-*xoc_3982* RNA interaction *in vivo*. All mutations were constructed in the *Xoc_3982*::*gfp* fusion strain, and asterisks denote point mutations. Lanes: 1, deletion mutant ΔXonc3711; 2, Xoc_3982::gfp fusion strain (labeled ‘Xonc3711’); 3, Xonc3711*; 4, ΔXonc3711-3982*; 5, 3982*; and 6, Xonc3711*-3982*. Upper three panels show northern blots using Xonc3711 or Xonc3711*, *xoc_3982*, and 5S rRNA as probes. The lower two panels show Xoc_3982^GFP^ or Xoc_3982*^GFP^ protein levels as determined by immunoblotting with mouse anti-GFP antisera; RNAP was used as a loading control. (b) RNase E is essential for *xoc_3982* repression by Xonc3711. Upper two panels show northern blot analysis of Xonc3711 expression in the ΔRNaseEC mutant, the wild-type BL256, and the ΔXonc3711 and ΔRNaseECΔXonc3711 mutants. The lower two panels show western blot analysis of Xoc_3982^GFP^ production. GFP fusion proteins were detected using anti-GFP antisera. Values above each band represent band intensity and were calculated using Image J software. Band intensity in the first lane was normalized as 100. ND, not detected.

We validated the Xonc3711-Xoc3982 interaction *in vivo* with compensatory point mutations. In mutant Xonc3711*, nucleotides UGC were mutated to CAA, whereas nucleotides ACG were mutated to GTT in xoc_3982* (Fig 2A). As predicted, Xonc3711 repressed *xoc_3982* expression relative to the deletion mutant (Fig 3, lanes 1 and 2); however, Xonc3711* was impaired in its ability to repress *xoc_3982* relative to the WT (Fig 3A, lane 3). Expression of xoc_3982* was only slightly reduced in the wild-type containing Xonc3711 (Fig 3A, lane 3); however, a high level of *xoc_3982* repression was observed when the compensatory mutations in Xonc3711* and xoc_3982* interacted (Fig 3A, lane 6). Collectively, these results suggest that Xonc3711 pairs with the *xoc_3982* mRNA CDS to inhibit *xoc_3982* expression.

### RNase E is required for Xonc3711-dependent degradation of *xoc_3982*

Ribonuclease RNase E is a critical enzyme in sRNA processing and turnover [23]. To better understand the sRNA-dependent degradation of *xoc_3982* mRNA, we examined the role of RNase E in Xonc3711-*xoc_3982* decay. The C-terminus of RNase E forms a scaffold and is involved in RNA degradation [24]; thus a C-terminal deletion in RNase E was constructed in strain BLS256 and was designated ΔRNaseEC (S1 Table). A second mutation was generated in this mutant by deleting Xonc3711, resulting in the double mutant ΔRNaseECΔXonc3711. The ΔRNaseEC strain showed significant upregulation in Xoc_3982::GFP protein levels when compared with the WT (Fig 3B, lanes 1 and 2), and Xoc_3982::GFP protein levels were only slightly higher in the double mutant strain (lane 4). These results indicate that RNase E contributes to the Xonc3711-mediated degradation of *xoc_3982*.

### Xonc3711 mutant shows decreased tolerance to oxidative stress

**Fig 4:**
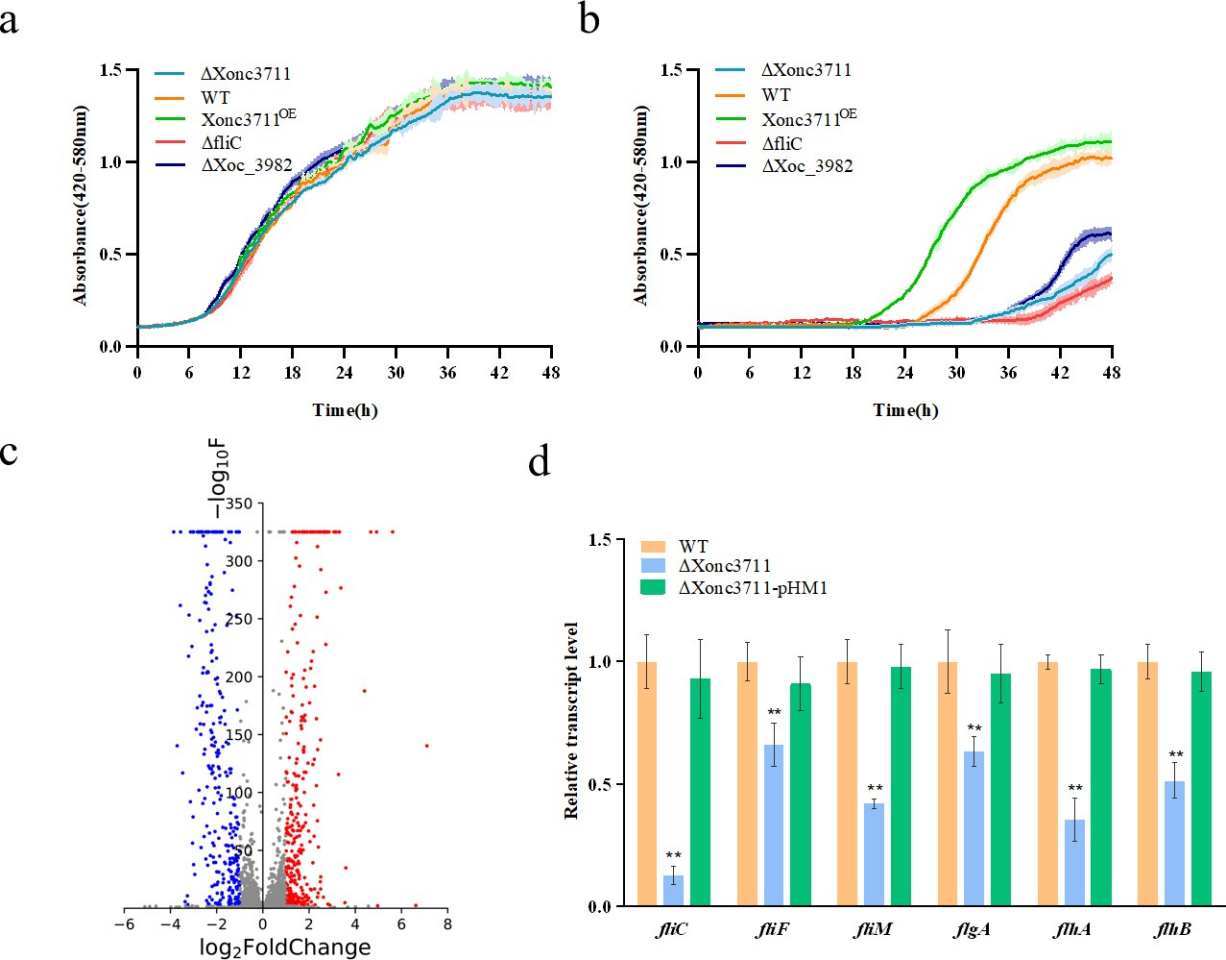
Mutations in Xonc3711, *xoc_3982*, and *fliC* decrease tolerance to oxidative stress. Panels: (a) growth in NB and (b) NB supplemented with 0.1 mM H_2_O_2_. Strains evaluated for growth included wild-type (WT) *Xoc* BL256, *ΔXonc3711*, Xonc3711^OE^, *ΔfliC*, and *ΔXoc_3982* in NB. Strains were grown in quadruplicate to the mid-exponential phase, diluted to OD_600_ = 0.1, and transferred to fresh NB or NB with 0.1 mM H_2_O_2_; growth was measured every 15 min for 48 h in a Bioscreen C apparatus at 28°C. Error intervals (shaded regions) indicate means ± SE (*n* = 4). (c) Volcano plot showing the FDR *P* values and fold-change of ΔXonc3711 versus the WT by RNA-Seq. Red and blue dots indicate upregulated and downregulated genes, respectively, with FDR *P* < 0.01. (d) Expression levels of *fliC*, *fliF*, *fliM*, *flgA*, *flhA*, and *flhB* in WT, ΔXonc3711, and ΔXonc3711-pXonc3711. Expression levels of flagella-related genes were calculated relative to *rpoD* using the ΔΔCT method, where CT is the threshold cycle. Four independent biological replicates were carried out in this experiment.

Xonc3711 expression was measured in *Xoc* BLS256 at 0, 7, 15, and 45 min after exposure to 0.1 mM H_2_O_2_ (S1C Fig). Xonc3711 transcript levels were highest at the 15 min time point, indicating that Xonc3711 expression was induced by oxidative stress. To further investigate the potential role of Xonc3711 in oxidative stress, growth of selected strains was compared in the presence and absence of 0.1 mM H_2_O_2_ in NB medium (Fig 4). Strains grown in NB without H_2_O_2_ showed similar growth patterns (Fig 4A); however, a delayed lag phase of approximately 8 h was observed in the ΔXonc3711 and ΔXoc_3982 strains grown in NB supplemented with 0.1 mM H_2_O_2_ when compared with the WT (Fig 4B). Pairwise comparisons of OD values for each strain and growth condition were analyzed using the Kolmogorov-Smirnov test against the values obtained for the WT. Strain Xonc3711^OE^ showed a high tolerance to 0.1 mM H_2_O_2_ (*P* < 0.01), whereas ΔXonc3711 showed a significantly reduced tolerance to oxidative stress relative to the WT (*P* < 0.01, Fig 4B). The ΔXoc_3982 mutant was also impaired in oxidative stress tolerance relative to the WT (*P* < 0.01, Fig 4B). These results suggest that the sRNA Xonc3711 interacts with the DNA-binding protein Xoc_3982 to help *Xoc* BLS256 adapt to oxidative stress.

### Xonc3711 impacts flagella structure and reduces biofilm formation

**Fig 5:**
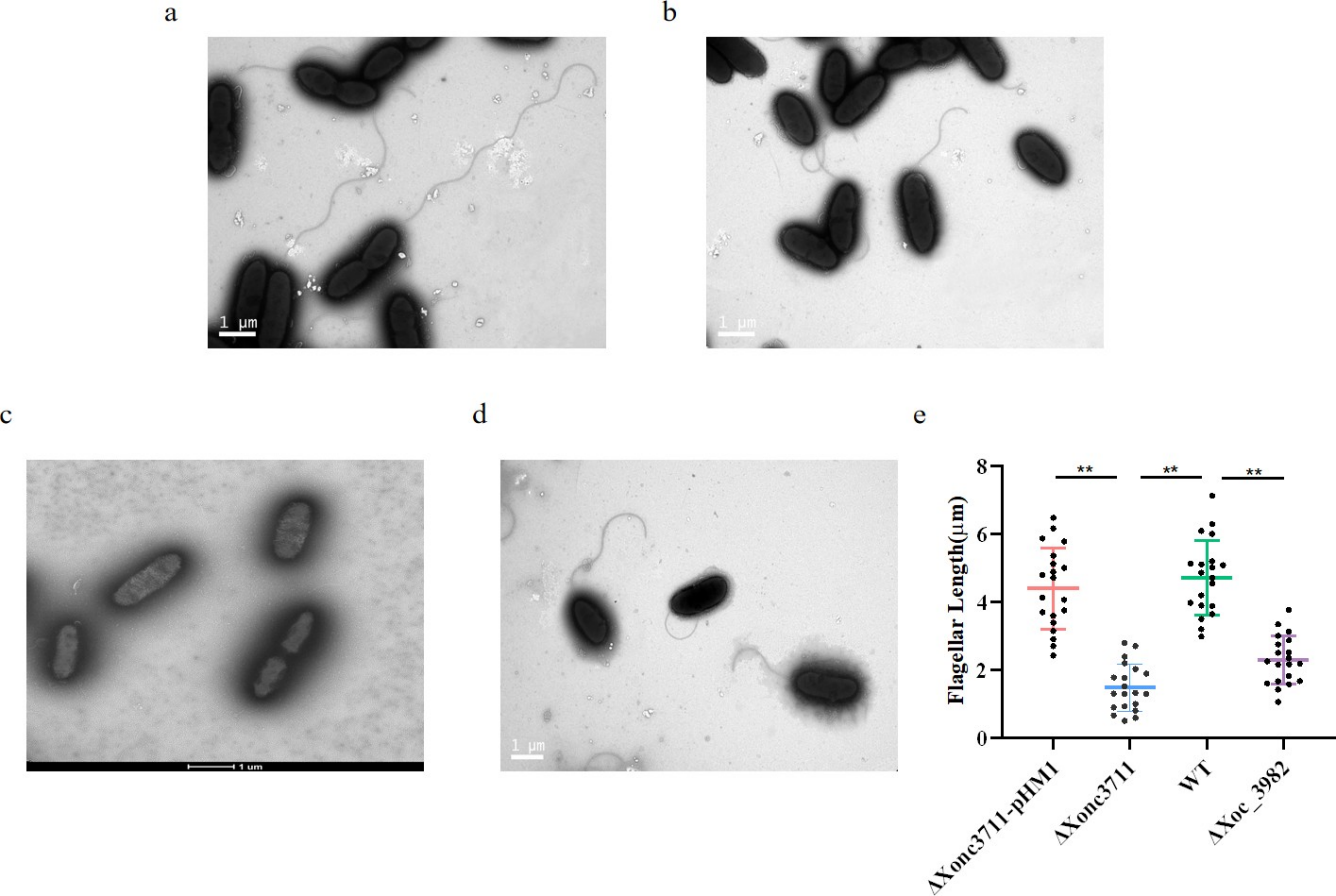
Mutations in Xonc3711 and *xoc_3982* reduce flagella numbers and length. Ultrastructure of (a) wild-type *Xoc* BL526; (b) ΔXonc3711, (c) ΔfliC, and (d) ΔXoc_3982. (e) Length of flagella (μM) produced by WT, ΔXonc3711, ΔXonc3711-pXonc3711, and ΔXoc_3982. Flagella were randomly measured from 20 parallel views by transmission electron microscopy. **, significant at *P* < 0.01, Wilcoxon rank-sum test.

RNA-seq was used to compare WT and ΔXonc3711 to further understand the involvement of sRNA Xonc3711 in oxidative stress tolerance. Prior to comparing RNA-seq profiles, reproducibility was evaluated in two replicate experiments using pairwise linear correlation analysis. The correlation coefficients (*r*) between the two replicate experiments were 0.999 and 0.997, indicating reproducibility of the RNA-seq data under the experimental conditions. Based on a stringent FDR (<0.01) as a cutoff, a large number of genes were downregulated in ΔXonc3711, including genes involved in flagella assembly, basal body formation, flagella motor, and T3SS-related genes (S2 Fig). Multiple genes involved in flagella synthesis and assembly, including *fliC*, *fliF*, *fliM*, *flgA*, *flhA*, and *flhB*, were downregulated in ΔXonc3711 as compared to the WT (Fig 4D). Interestingly, the expression of *fliC*, which encodes the flagellar filament structural protein, was approximately 8-fold lower in ΔXonc3711 as compared to the WT (Fig 4D). *In vitro* growth curves revealed that the *fliC* mutant was less tolerant to H_2_O_2_ than the wild-type, and growth of the *fliC* mutant was similar to ΔXonc3711 during oxidative stress (Fig 4B). These results indicated that Xonc3711 has an impact on the structure of flagella. The effect of Xonc3711 on flagella was assessed by comparing the ultrastructure of selected strains via high-resolution transmission electron microscopy (TEM). The mutants ΔXonc3711 and ΔXoc_3982 produced fewer flagella than the WT (Fig 5A, 5B and 5D). Ten fields of view were randomly selected from the WT and ΔXonc3711 and used to compile statistical differences in flagellar length (Fig 5A and 5B). The results showed that flagella in ΔXonc3711 were significantly shorter than the WT (Fig 5E). A *fliC* deletion mutation (Δ*fliC*) was used as a control and was devoid of flagella as expected (Fig 5C). Interestingly, the phenotype of the Xoc_3982 mutant with respect to flagella was similar to ΔXonc3711; in other words, the ΔXoc_3982 mutant produced fewer, shorter flagella than the WT (Fig 5D and 5E). These results suggest that mutations in both Xonc3711 and *xoc_3982* impact flagella synthesis and structure.

**Fig 6:**
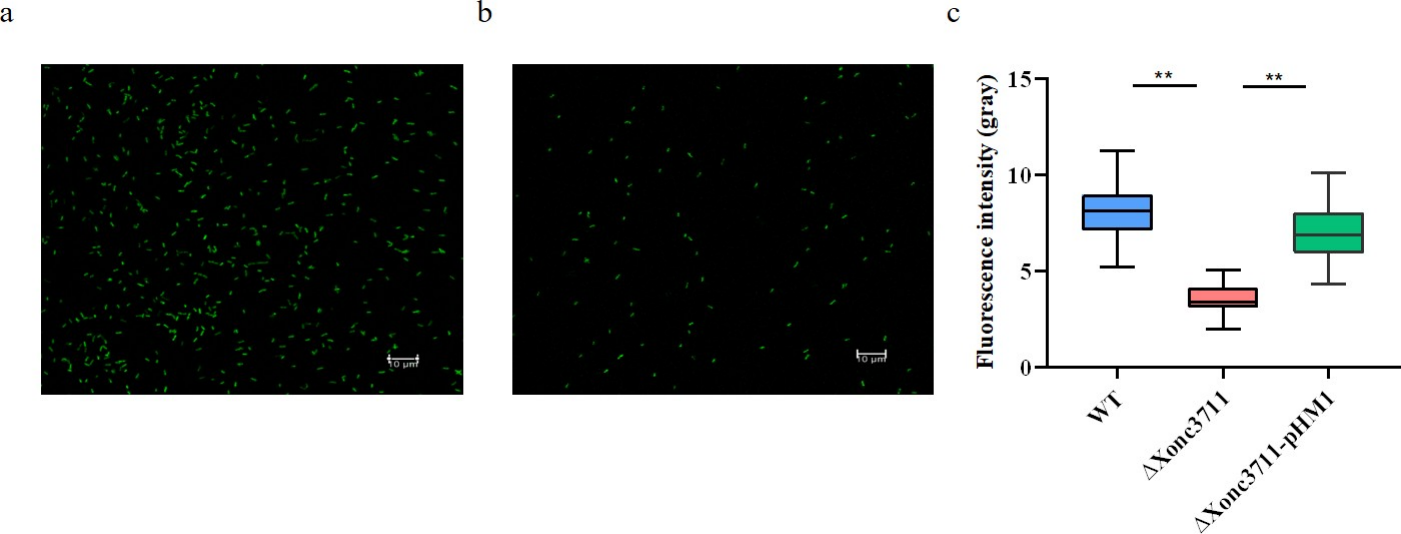
Xonc3711 contributes to biofilm formation. Confocal laser scanning microscopy of fluorescence in (a) GFP-labeled wild-type *Xoc* BL256 and (b) GFP-labeled ΔXonc3711. (c) Fluorescence in GFP-labeled *Xoc* WT, ΔXonc3711, and the complemented strain, ΔXonc3711-pXonc3711. Bacteria were grown under static conditions at 28°C for 96 h on glass coverslips. Biofilms were fixed, and fluorescence intensity was measured from 20 parallel replicates. **, *P*<0.01, Wilcoxon rank-sum test.

Previous studies have demonstrated that flagella-driven motility facilitates the formation of biofilms [25], which contribute to oxidative stress tolerance [26, 27]. Our results indicate that Xonc3711 contributes to both oxidative stress and motility; thus, we measured biofilm formation using a confocal laser scanning microscope (CLSM) and 3D serial layer scanning. Mutant ΔXonc3711 was impaired in its ability to adhere to glass surfaces and showed reduced fluorescence when compared to the WT (Fig 6 and S1 Video); these results confirm a relationship between sRNA Xonc3711 and *Xoc* motility and biofilm formation.

### Xonc3711 overexpression contributes to virulence

**Fig 7:**
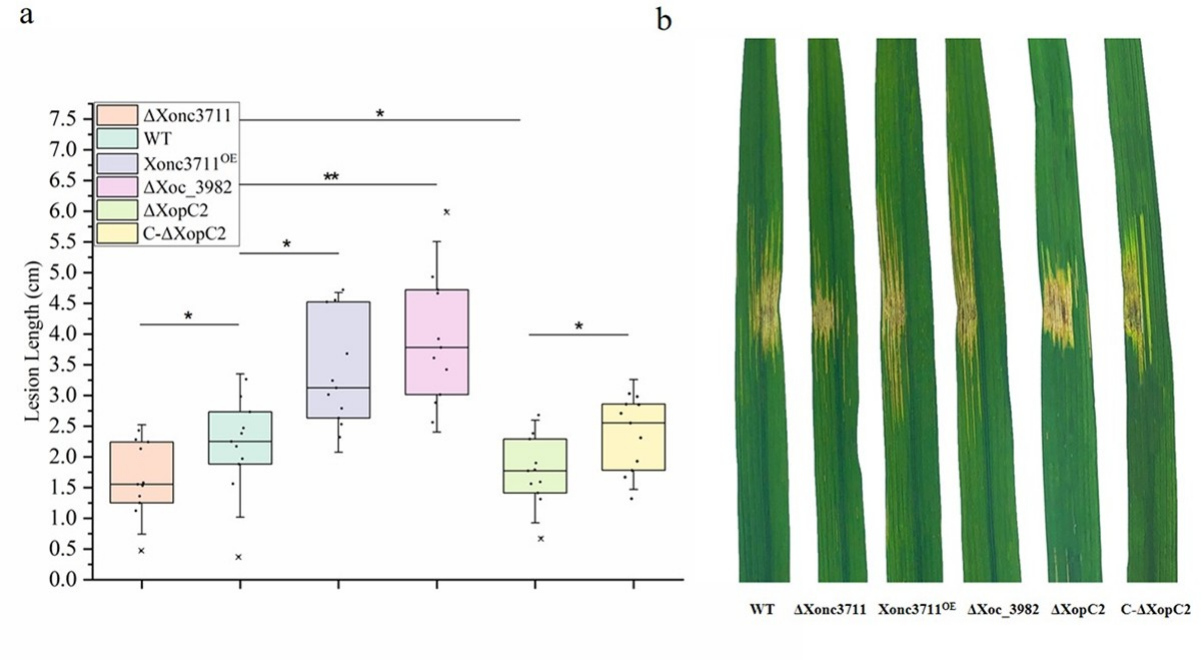
Virulence and in *planta* growth of *Xoc* strains in rice cv. Yuanfengzao. Virulence was assessed by inoculating six-week-old susceptible rice plants. (a) Leaves (*n* = 11) were inoculated with needleless syringes, and lesion lengths were evaluated 14 d after inoculation. Results indicate means ± SD. ANOVA was performed with Dunnett’s multiple-comparison post-hoc correction as compared with the WT (*, *P* < 0.05; **, *P* < 0.01). (b) Symptoms on rice leaves inoculated with *Xoc* WT, ΔXonc3711, Xonc3711^OE^, Xoc_3982, ΔXopC2, and C-ΔXopC2.

Bacterial biofilms are generally more resistant to antimicrobial agents and host defense systems than individual cells; furthermore, bacterial biofilms may exhibit stronger virulence than cells in a planktonic state [28]. To evaluate the potential contribution of Xonc3711 to virulence, leaves of six-week-old rice cv. Yuanfengzao were inoculated with the WT, ΔXonc3711, ΔXoc_3982, and Xonc3711^OE^ (Fig 7A). At 14 d post-inoculation, lesions induced by Xonc3711^OE^ were significantly larger than those induced by the WT and ΔXonc3711 (Fig 7B). Interestingly, the ΔXoc_3982 mutant showed elevated virulence and produced slightly larger lesions than the WT and ΔXonc3711. Thus, our results suggest that Xonc3711 contributes to virulence in in *Xoc* BLS256.

### Xoc_3982 directly regulates the effector encoded by *xopC2*

**Fig 8:**
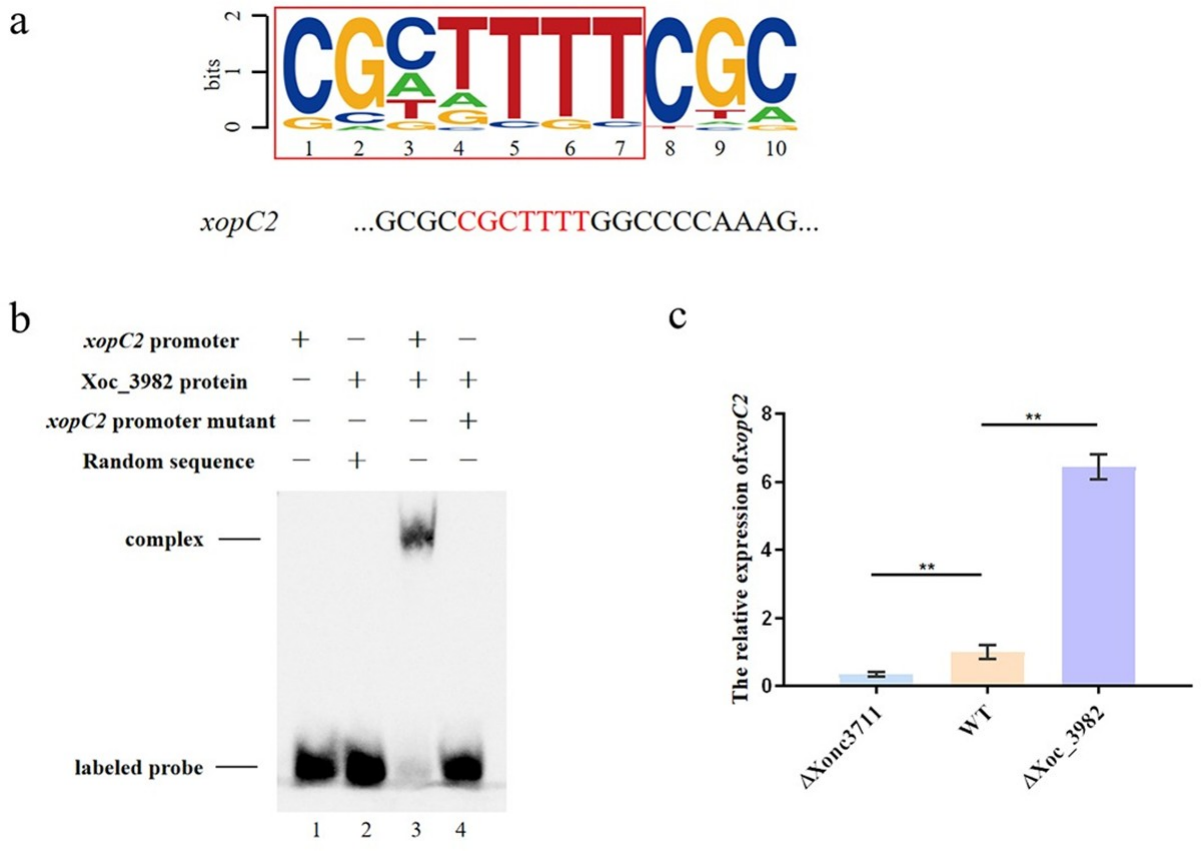
Xoc_3982 recognizes a conserved motif and regulates effector gene *xopC2*. (a) A potential Xoc_3982 binding motif was identified by MEME analysis of ChIP-seq peak regions. Representative sequences potentially bound by Xoc_3982 are listed. A conserved sequence in the promoter of the five genes is shown in red. (b) Interaction of the wild-type *xopC2* promoter, the Xoc_3982 protein, and a *xopC2* promoter mutant in electrophoretic mobility shift assays. Lane 1, 3’-biotinylated *xopC2* promoter; lane 2, Xoc_3982 and a 3’-biotinylated random sequence (5’-TGTACAGTGATCAGTACAGG-3’); lane 3, 3’-biotinylated *xopC2* promoter and Xoc_3982; lane 4, mutated *xopC2* promoter and Xoc_3982. (c) qRT-PCR analysis of *xopC2* expression levels in ΔXonc3711, WT BL256 and Δxoc_3982.

The Xoc_3982 protein was analyzed at the NCBI Conserved Domain Database (https://www.ncbi.nlm.nih.gov/cdd), and the results indicated that Xoc_3982 was a potential DNA-binding protein with relatedness to DNA modification/repair proteins in the radical SAM family (S3 Fig). The xoc_3982::GFP fusion X+3 (pKMS1::X+3, S1 Table) was introduced into *Xoc* BLS256 and used in a ChIP-seq assay to identify genes regulated by Xoc_3982. The results showed that potential targets of Xoc_3982 included *hemF*, *xdhC*, *xpsE*, *xopC2*, and *mutM*; these genes contained a conserved sequence, 5’-CGCTTTT-3’, which was identified by MEME analysis as a putative Xoc_3982 binding site (Fig 8A). We were particularly interested in *xopC2*, which encodes a T3SS effector that has been identified in a number of xanthomonads, including *Xoc BLS256* [29–32] and was shown to function in the virulence of *X*. *axonopodis* pv. *punicae* [33]. RNA-seq data indicated that the expression of *xopC2* was downregulated in the ΔXonc3711 mutant as compared to the wild-type BL526. The putative Xoc_3982 binding site was located in the *xopC2* promoter at -334 to -327 with respect to the translational start site. EMSA confirmed that Xoc_3982 interacted with the *xopC2* promoter, and the interaction was disrupted when the *xopC2* promoter was mutated (Fig 8B). The relative expression of *xopC2* was significantly higher in the ΔXoc_3982 mutant than the WT, which suggests that Xoc_3982 is a negative regulator of *xopC2* (Fig 8C). Furthermore, the ΔXopC2 strain was downregulated in virulence when compared to the WT (Fig 7), which indicated that *xopC2* contribute to the virulence in *Xoc* BLS256.

## Discussion

In this report, sRNA Xonc3711 was shown to control expression of *xoc_3982*, which encodes a DNA-binding protein in *Xoc* BLS256. Xoc_3982 repressed the expression of the T3SS effector encoded by *xopC2*, which suggests that Xoc_3982 functions as a transcriptional repressor. It is important to note that sRNAs can positively or negatively modulate transcriptional regulators [34]; for example, the sRNAs CsrB and CsrC in *E*. *coli* sequester the translational repressor CsrA, which impacts biofilm formation [35]. Although the precise mechanisms are unclear, sRNA Xonc3711 modulates multiple traits in *Xoc* including the formation of flagella and biofilms; this suggests that Xonc3711 regulates genes that interact with the external environment.

Target sites of small RNAs are often present in the 5’ UTR of the target gene; however, exceptions exist and bacterial sRNAs have been identified that lack obvious binding sites in the 5’ UTR of the target gene [36, 37]. For example, the *Salmonella typhimurium* sRNA MicC targets the *ompD* mRNA within its CDS [38]. Similarly, we show that Xonc3711 targets the *xoc_3982* mRNA within the CDS (Fig 2B and 2C). Mutational analysis showed that the regulation of *xoc_3982* is direct and requires an antisense interaction between Xonc3711 and *xoc_3982* mRNA (Fig 2A); this was confirmed by EMSA (Fig 2D). Another important feature of sRNA Xonc3711 is an A/U-rich motif that could bind the RNA chaperone Hfq for stabilization and base-pairing [39]. Although the precise nucleotides in Xonc3711 that interact with Hfq were not identified in this study, Xonc3711 and Hfq interacted in gel shift assays (Fig 1A).

Seed-borne pathogens like *Xoc* are exposed to reactive oxygen in the natural environment and inside the plant host during the defense response [10]. Liang et al. [19] previously reported that the expression of over 20 genes, including superoxide dismutase, was regulated in the sRNA-Xoo1 mutant; these findings indicated that sRNA-Xoo1 is likely involved in oxidative stress tolerance in *Xoo* PXO99. To deal with oxidative stress, bacterial pathogens often deploy enzymes that either tolerate or scavenge ROS. We recently demonstrated that adenosine-to-inosine (A-to-I) RNA editing in *Xoc* increased tolerance to H_2_0_2_ [20]. A-to-I editing in the target *fliC* caused structural changes in flagella that increased biofilm formation and ultimately improved ROS tolerance [20]. A number of studies have shown that sRNAs can also regulate tolerance to oxidative stress in prokaryotes [22]. In the present study, we used a genetic approach to show that sRNA Xonc3711 contributes to ROS tolerance in *Xoc* BLS256.

RNA-seq showed that multiple flagella-related genes were downregulated in ΔXonc3711 (Figs 4D and S2); however, our analysis failed to identify a Xonc3711 target that was involved in flagella biosynthesis or regulation. Flagellar-driven motility is critical for biofilm development in many pathogens [25], and biofilm formation is associated with increased adhesion of bacteria to surfaces and improved stress resistance [40, 41]. We measured biofilm formation by TEM, and discovered that adherence of ΔXonc3711 to glass surfaces was severely inhibited as compared to the WT (Fig 6 and S1 Video). Thus, it seems likely that the reduced biofilm formation by the ΔXonc3711 mutant resulted in decreased tolerance to oxidative stress.

Exopolysaccharides, degradative enzymes, and toxins all contribute to virulence in *X*. *oryzae* [42–44]. Most phytopathogenic xanthomonads secrete effector proteins via the T3SS to suppress the defense response. The effectors that are designated as *Xanthomonas* outer proteins (Xops), are known to be key factors required for bacterial growth and colonization in distinct eukaryotic host [45]. In this study, we also established an important role for Xonc3711 in *Xoc* virulence and demonstrated that the DNA-binding protein *xoc_3982*, the target of Xonc3711, negatively regulates *xopC2* expression (Fig 8C). A genetic approach was then used to show that *xopC2* contributes to lesion size in Xoc BL526 (Fig 7B). Efforts are underway to identify additional genes regulated by Xoc_3982 to fully understand its role in bacterial metabolism and virulence.

Effector proteins encoded by *hrp* (*h*ypersensitive *r*eaction and *p*athogenicity) gene clusters are important virulence factors in pathogens. HrpX, a key regulator of *hrp* genes, regulates the expression of effector genes at a conserved plant-inducible promoter (PIP)-box in the effector promoter region [46]. The PIP-box is a conserved cis-element and its sequence, TTCGB-N_15_-TTCGB (B stands for any base except A), is generally located about 30 bp upstream of the effector gene start codon [46]. ChIP-seq data revealed the potential Xoc_3982 binding site as 5’-CGCTTTT-3’ (region -327 to -334 with respect to the translational start site of *xopC2*); however, the *xopC2* promoter region does not contain a PIP-box. In this regard, *xopC2* is similar to other *xop* genes that lack PIP boxes but maintain regulation by HrpX [47]. We did not confirm a role for HrpX in *xopC2* regulation; however, this has been reported for *xopC* in *X*. *campestris* pv. *vesicatoria* [47].

**Fig 9:**
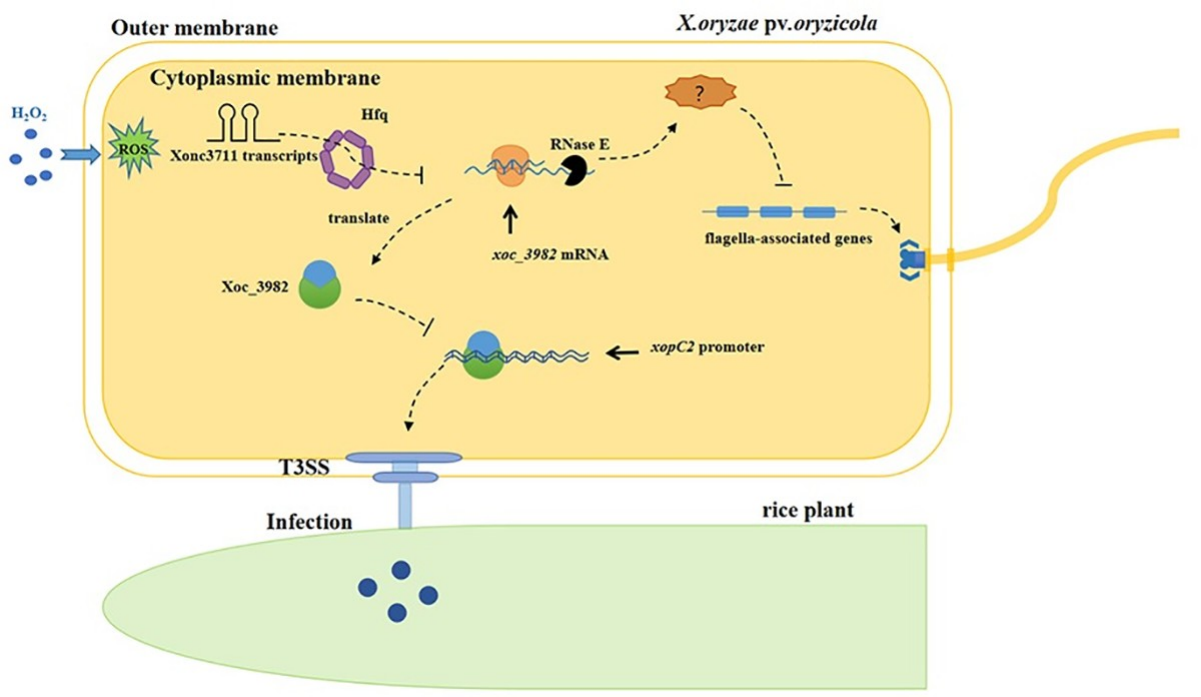
Proposed model for Xonc3711-mediated regulation of flagella production and virulence. Additional copies of the sRNA Xonc3711 transcript are produced during oxidative stress. Hfq-dependent Xonc3711 targets *xoc_3982* mRNA, and Xonc3711-induced degradation of *xoc_3982* mRNA is dependent on Rnase E; this results in the expression of flagella-associated genes though an unknown molecule. The DNA-binding protein Xoc_3982 interacts with the promoter of *xopC2*, repressing its transcription. When repression is lifted, XopC2 traverses the T3SS, enters the plant cell, and interacts with proteins that enhance the infection process.

This study provides insight into RNA-mediated regulation of environmental signaling in bacterial physiology and pathogenesis (Fig 9). Xonc3711 base pairs within the *xoc_3982* CDS to inhibit translation, which is relatively rare for sRNAs [48, 49]. Xonc3711 contributes to biofilm formation and improves oxidative stress tolerance in *Xoc* BLS256. Based on ChIP-seq data, the DNA-binding protein Xoc_3982 was found to bind to the promoter region of *xopC2*, a T3SS effector that has been implicated in virulence in some xanthomonads [33]. The identification of other Xonc3711 targets will be helpful in understanding the biological circuitry regulated by sRNAs in phytopathogenic *Xanthomonas* spp.

## Materials and methods

### Strains, plasmids and primers

The bacterial strains and plasmids used in this study are described in S1 Table. Primers used for the construction of mutant strains, plasmids and DNA templates are provided in S2 Table.

### Growth conditions

*Escherichia coli* strains were cultured in Luria-Bertani (LB) medium at 37°C. *Xoc* BLS256 and derivative strains were grown in nutrient broth (NB) or NB containing 1.5% (w/v) agar (NA) as described previously [20]. Antibiotics were added to media in the following final concentrations (μg/mL): ampicillin, 100; cephalexin, 40; kanamycin, 25; and spectinomycin, 50. Expression from the P_*lac*_ promoter was induced by addition of 1 mM IPTG [20].

Seeds of rice cv. Yuanfengzao were obtained from the International Rice Research Institute and cultivated at Shanghai Jiao Tong University as described.

### Construction of deletion, point and overexpression mutants

Bacterial mutant strains were generated as described by Baba with minor modifications [50]. Two fragments flanking the target gene were amplified from the chromosomal DNA of *Xoc* BLS256 using *Pfu* polymerase (TransGen Biotech, Beijing, China) and the primers described in S2 Table. The PCR products were digested, subcloned into the suicide vector pKMS1 [51], and introduced into bacteria by electroporation (Bio-Rad Laboratories Inc., Hercules, CA, USA) with kanamycin selection. A single transformant with kanamycin resistance was selected, cultured for 8 h in NB, and inoculated as 10-fold dilutions to NA with 15% sucrose to obtain sucrose-insensitive clones. For site-directed mutagenesis, plasmids were modified with the Fast Mutagenesis System (Transgen Biotech, Beijing, China) to obtain clones containing point mutations (Xonc3711*, 3982*, xopC2*; S1 Table).

To obtain the Xonc3711 overexpression mutant (Xonc3711^OE^), the full-length corresponding gene was amplified, and the fragment was cloned into pHM1 with the *lac* promoter. The recombinant plasmid was transferred into WT by electroporation, and transformants were screened on NA plates supplemented with spectinomycin.

### Bacterial growth and gene expression in response to oxidative stress

The optical density of bacterial solutions was measured with a Bioscreen C (Labsystem, Helsinki, Finland). Individual wells of a microtiter plate containing 99 μL of NB or LB broth with or without 0.1 mM H_2_O_2_ were inoculated with 1 μL of overnight suspensions of *Xoc* (1 × 10^9^ CFU/mL). OD values at 420–580 nm were obtained at 15 min intervals over a 48 h period with constant agitation at 28°C. Viable cell counts in the presence and absence of H_2_O_2_ were determined as described previously [20]. All experiments were performed in quadruplicate, and the Kolmogorov–Smirnov test was used to evaluate significance.

Assays for resistance to H_2_O_2_ were performed as described previously [52]. Briefly, *Xoc* strains were cultured to the mid-log phase (OD_600_ = 1.0 ~ 1.2) and exposed to 0.1 mM H_2_O_2_ at 28°C; aliquots were removed at 0, 7, 15, and 45 min and pelleted by centrifugation at 4°C. Pellets were washed twice in cold PBS, and the total RNA was immediately extracted using the RNeasy Protect Bacteria Mini Kit (Qiagen) as recommended. Two biological replicates were used in this experiment.

### Visualization of biofilms and flagella

Biofilm production by *Xoc* was visualized using GFP-labeled strains as described previously [20, 53]. Protocols used for observing biofilms by confocal microscopy have been described [20]. Images, surface topographies and 3D architectures were processed with the Leica Application Suite X (v. 3.4.2.18368).

TEM was used to detect the formation of flagella by *Xoc* strains. Samples were mounted on carbon-coated grids for 1 min, washed with deionized water and negatively stained with 3% (w/v) phosphotungstic acid for 30 s. A Talos F200 transmission electron microscope (Thermo Fisher Scientific, USA) was used to acquire images at 120 kV.

### mRNA purification and cDNA synthesis

Samples of total RNA (10 μg) were treated with the MICROBExpress Bacterial mRNA Enrichment kit (Ambion) and RiboMinus Transcriptome Isolation Kit (Bacteria) (Invitrogen) as recommended by the manufacturers’ instructions. Total RNA samples were resuspended in 15 μL of RNase-free water, chemically fragmented to 200–250 bp and used to generate cDNA with Magic 1st cDNA Synthesis Kit (Magic-Bio, China) as described previously [52].

### RNA sequencing and analysis

The Illumina Paired End Sample Prep kit was used to create a RNA-Seq library as described [52]. After removing low quality reads and adaptors, RNA-Seq reads were aligned to the corresponding *Xoc* BLS256 genome using Tophat 2.0.7 [54]as described previously [20]. Differentially expressed genes (FDR value < 0.01) were selected for further analysis. Heatmaps were generated using Cluster 3.0 and Treeview 1.1.6 based on reads per kb of transcript per million mapped reads (RPKM) values [55, 56].

### Chromatin immunoprecipitation sequencing (ChIP-seq)

ChIP-Seq libraries were prepared and sequenced as described previously [57]. Briefly, DNA fragments (200–500 bp) were selected using solid phase reversible immobilization beads and amplified by PCR for 15 cycles after repair and adaptor ligation steps. Validation of libraries was performed using the Bioanalyzer 2100 (Agilent, Santa Clara, CA, USA) and Qubit fluorometer (Invitrogen, Carlsbad, CA, USA). Sequencing was performed using the HiSeq 2000 system (Illumina, San Diego, CA, USA), and Trimmomatic v. 0.38 [58] was used to remove low-quality reads. Clean reads were mapped to the *xoc_3982*+3::*gfp* genome using the Burrows-Wheeler Aligner v. 0.7.15 [59], and potential PCR duplicates were removed using SAMtools v. 1.3.1. [60] Peaks were called using model-based analysis of ChIP-Seq (MACS) v. 2.1.1.20160309 as described previously [61]. Motifs were detected with HOMER (Hypergeometric Optimization of Motif EnRichment v. 3; http://homer.ucsd.edu/homer), and the EasyGO tool (http://bioinformatics.cau.edu.cn/easygo) was used for gene ontology analysis as described [61]. ClusterProfiler (www.bioconductor.org/packages/release/bioc/html/clusterProfiler.html) was used for KEGG enrichment analysis (Kyoto Encyclopedia of Genes and Genomes, http://www.genome.jp/kegg/).

### In vitro synthesis and labeling of RNA

*Xoc_3982* mRNA and Xonc3711 sRNA were prepared using 5 μg of DNA that was generated by PCR with primers F/R-T7Xoc_3982 and F/R-T7Xonc3711 (S2 Table) and the Megascript T7 Transcription Kit (Ambion, Austin, TX, USA). The MAXIscript T7 In Vitro Transcription Kit (ThermoFisher, USA) was used to synthesize RNA from DNA templates; and the RNA transcripts were purified with the MEGAclear Transcription Clean-Up Kit (ThermoFisher,USA). A biotinylated nucleotide was added to the 3’ termini of the synthesized RNA molecules using the Pierce RNA 3’ End Biotinylation Kit (ThermoFisher, USA) as recommended by the manufacturer.

### Electrophoretic mobility shift assays

The Hfq and Xoc_3982 proteins were expressed and purified using the intein-based Impact Kit (New England Biolabs, USA) as described [9]. Binding reactions were conducted in 10 μl volumes with the LightShift Chemiluminescent RNA EMSA Kit (ThermoFisher, USA); reactions were incubated at 37°C for 20 min, and 5 μl of loading buffer (50% glycerol) was then added. The interaction of Hfq and Xonc3711 sRNA was conducted in 1× binding buffer with 3’-biotinylated Xonc3711 sRNA. The interaction of sRNA Xonc3711-with Xoc_3982 mRNA was investigated using EMSA as described previously [9]. Samples were separated in 5% nondenaturing polyacrylamide gels in 0.5× TBE at 4°C and visualized by phosphoimaging on a ChemiScope 3000 mini (CLiNX, Shanghai, China).

### Northern blot analysis

Total RNA was purified from *Xoc* BLS256 liquid cultures (OD_600_ = 1.0) using the EasyPure RNA Kit (Transgen Biotech, Beijing, China). RNA (10–20 μg) was separated in 1% agarose gels containing 25 mM guanidium thiocyanate, transferred to Hybond N+ nitrocellulose membranes (Merck Millipore, USA), and cross-linked to membranes by UV radiation. Probes were 5’-labeled with digoxygenin (DIG). Membranes were prehybridized for 10 min at 42°C, and then incubated with labeled probes overnight. Membranes were then rinsed, dried and visualized by phosphorimaging on a ChemiScope 3000 mini (CLiNX, Shanghai, China) as described previously [13].

### Western blot analysis

Samples were spun and cell pellets were re-suspended in 1×SDS loading buffer (3% SDS, 10% glycerol, 50 mM Tris–HCl pH 6.8, 0.1% bromophenol blue, 12.5 mM EDTA, 100 mM DTT) to a concentration of approximately 10^6^ cells/μl and boiled at 95°C for 10 min. Protein concentration was measured according to the manufacturer’s instructions for the BCA Protein Assay Kit (Solarbio, Beijing, China). Total proteins were separated by 12% sodium dodecyl sulfate-polyacrylamide gel electrophoresis (SDS-PAGE) and then transferred to polyvinylidene fluoride membranes (Merck Millipore, USA). GFP fusion proteins and RNA polymerase subunit α (RNAPα) were detected using antibodies directed against GFP (1:2000; mouse; Roche), anti-*E*.*coli* RNAP (1:2000; mouse; BioLegend, San Diego, CA, USA), and goat anti-mouse secondary antibodies conjugated with horseradish peroxidase (1:5000; Transgen Biotech). Signals were visualized using Western Lightning Plus-ECL (Edo Biotech) and detected with ChemiScope 3000 mini (CLiNX, Shanghai, China).

### Quantitative real-time PCR

qRT-PCR was conducted as described previously [20]. Gene expression was normalized relative to *rpoD* using the ΔΔCT method, where CT is the threshold cycle. Four independent biological replicates were included and analyzed using the Wilcoxon-Mann-Whitney test.

### Plant inoculations

Virulence assays were conducted with *Xoc* suspensions (OD_600_ = 0.8), which were inoculated to six-week-old seedlings of rice cv. Yuanfengzao with needleless syringes. Lesion lengths were measured 14 d after inoculation. Twelve or more leaves were inoculated and evaluated for each *Xoc* strain.

## Supporting information

S1 Fig Secondary structure, map location and response of Xonc3711 to oxidative stress. (a) Predicted secondary structure of Xonc3711. Predominant features in the secondary structure are labeled as follows: stems (S1–S6), bulges (B1- B5), loops (L1–L3), and single-stranded regions (SS1–SS2). (b) Schematic diagram showing location of *xonc3711* and flanking DNA. The light gray rectangle shows the location of *xoc3711*, which encodes a hypothetical protein. Dark gray rectangles indicate *tol* and *thiC*, which encode a putative regulatory protein and a thiamine biosynthesis protein, respectively. The light gray vertical arrow shows the location of sRNA Xonc3711. Sequence data generated in this study are deposited in NCBI under BioProject number PRJNA350867. (c) Relative expression of the SRNA Xonc3711 in *Xoc* BLS256 treated with 0.1 mM H_2_O_2_ at 0, 7, 15 and 45 min after cells reached OD_600_ = 1.0 in NB.
(TIF)

S2 Fig Schematic diagram of flagellar assembly. Genes in red-shaded rectangles were downregulated in ΔXonc3711 as compared to the wild-type in the RNA-seq data. Genes in green-shaded rectangles were not differentially expressed genes in the RNA-seq data. The model was derived from KEGG database (https://www.genome.jp/kegg-bin/show_pathway?xor02040).
(TIF)

S3 Fig Conserved domains in Xoc_3982 in Xoc BLS256 predicted by NCBI Conserved Domain Search. (TIF)

S1 Video 3D movie of biofilms produced by Xoc WT and the ΔXonc3711 mutant using confocal laser scanning microscopy. (WMV)

S1 Table Strains and plasmids used in this study. (DOCX)

S2 Table Primers used in this study. (DOCX)
