# Supplementary figures and images for: A key antisense sRNA modulates the oxidative stress response and virulence in Xanthomonas oryzae pv. oryzicola
Source: PLoS Pathog. 2021 Jul 23;17(7):e1009762. doi: 10.1371/journal.ppat.1009762 (PMC8336823; doi:10.1371/journal.ppat.1009762)

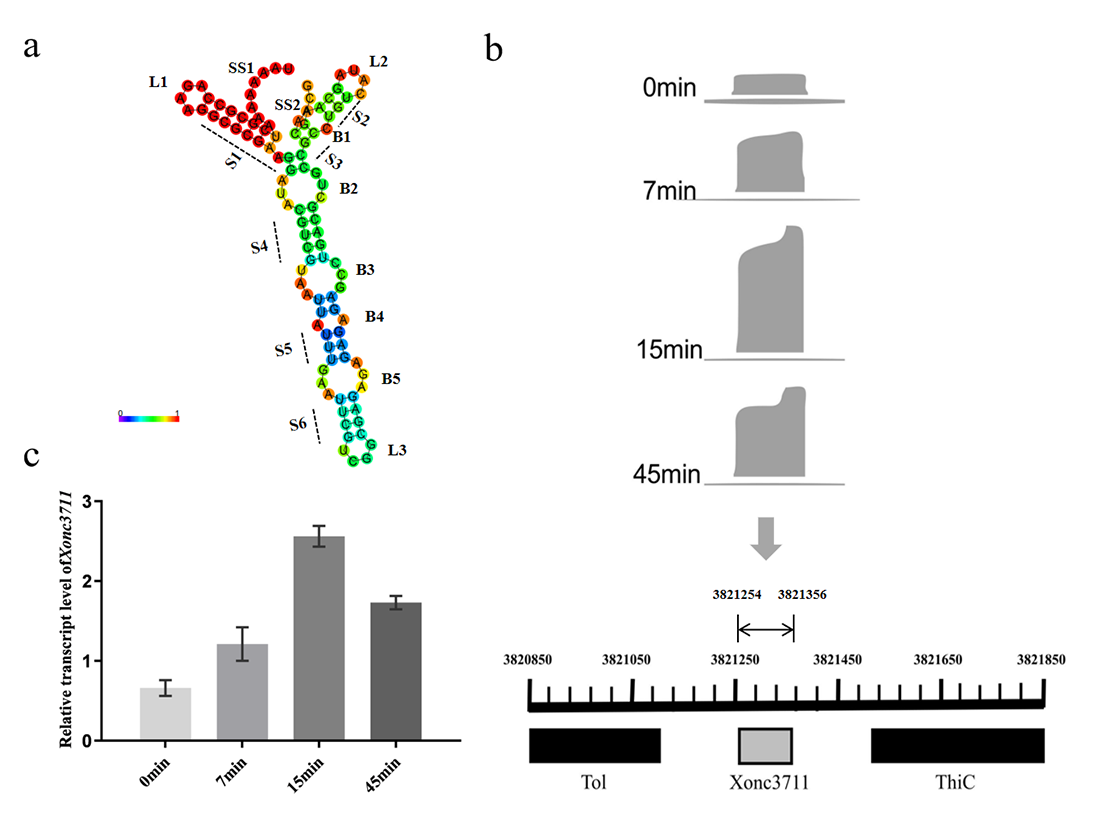

Supplement: S1 Fig — (a) Predicted secondary structure of Xonc3711. Predominant features in the secondary structure are labeled as follows: stems (S1–S6), bulges (B1- B5), loops (L1–L3), and single-stranded regions (SS1–SS2). (b) Schematic diagram showing location of xonc3711 and flanking DNA. The light gray rectangle shows the location of xoc3711, which encodes a hypothetical protein. Dark gray rectangles indicate tol and thiC, which encode a putative regulatory protein and a thiamine biosynthesis protein, respectively. The light gray vertical arrow shows the location of sRNA Xonc3711. Sequence data generated in this study are deposited in NCBI under BioProject number PRJNA350867. (c) Relative expression of the SRNA Xonc3711 in Xoc BLS256 treated with 0.1 mM H2O2 at 0, 7, 15 and 45 min after cells reached OD600 = 1.0 in NB. (TIF) [file ppat.1009762.s001.tif]

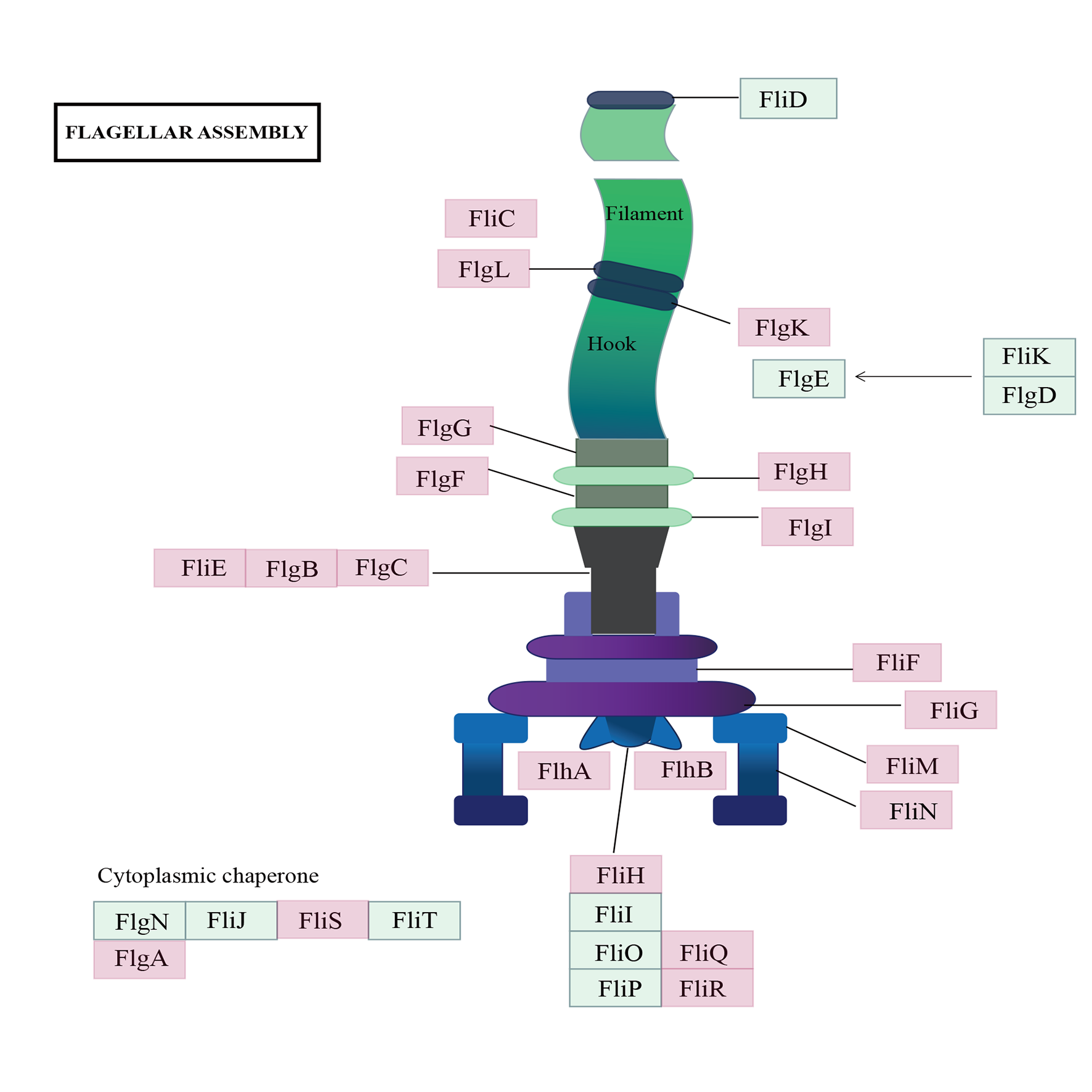

Supplement: S2 Fig — Genes in red-shaded rectangles were downregulated in ΔXonc3711 as compared to the wild-type in the RNA-seq data. Genes in green-shaded rectangles were not differentially expressed genes in the RNA-seq data. The model was derived from KEGG database (https://www.genome.jp/kegg-bin/show_pathway?xor02040). (TIF) [file ppat.1009762.s002.tif]

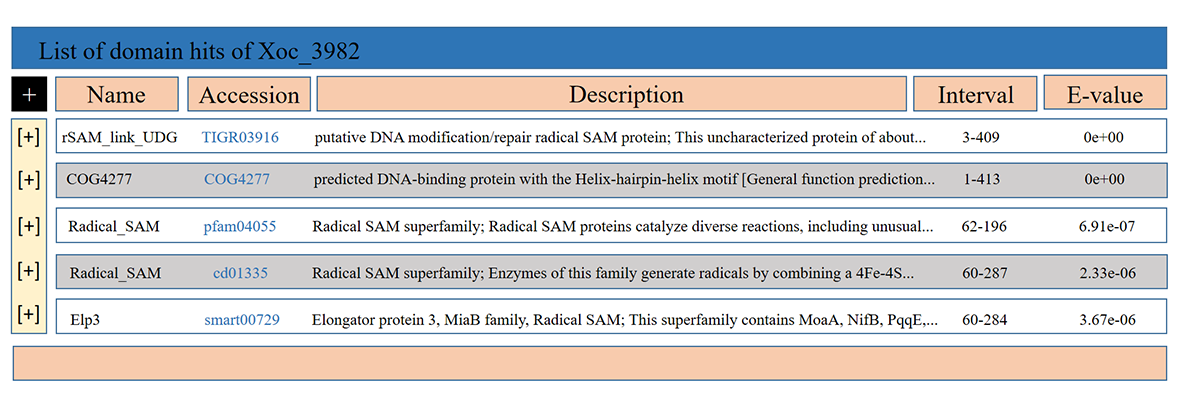

Supplement: S3 Fig — (TIF) [file ppat.1009762.s003.tif]
