# Supplementary material for: A key antisense sRNA modulates the oxidative stress response and virulence in Xanthomonas oryzae pv. oryzicola
Source: PLoS Pathog. 2021 Jul 23;17(7):e1009762. doi: 10.1371/journal.ppat.1009762 (PMC8336823; doi:10.1371/journal.ppat.1009762)
Supplement: S1 Table — (DOCX) [file ppat.1009762.s005.docx]

**S1 Table**: Strains and plasmids used in this study.

| **Strain or plasmid** | **Relevant characteristics** | **Source** | | | |
| --- | --- | --- | --- | --- | --- |
| Strains | | | | |  |
| *Escherichia coli* | | | | |  |
| DH5α | *F-*, *φ 80dlacZ ΔM15*, Δ (*lacZYA -argF*) *U169*, *deoR*, *recA1*, *endA1*, *hsdR17* (*rK-*, *mK+*), *phoA*, *supE44*, *λ-*, *thi -1*, *gyrA96*, *relA1* | | | Takara |  |
| BL21 | *F-*, *omp*, *ThsdSB(rB-mB-)*, *gal*, *dcm(DE3)* | | | Takara |  |
|  | | | |  |  |
| *Xanthomonas oryzae pv. oryzicola* | | | |  |  |
| BLS256 | Wild-type | | | [1] |  |
| X+3 | *Xoc_3982*+3::*gfp* | | | This study |  |
| X+1242 | *Xoc_3982*+1242::*gfp* | | | This study |  |
| ΔXonc3711 | sRNA Xonc3711 deletion mutant of BLS256 | | | This study |  |
| ΔHfq | *hfq* deletion mutant of BLS256 | | | This study |  |
| Δ3982 | *xoc_3982* deletion mutant of BLS256 | | | This study |  |
| Xoc_3982::gfp | *Xoc*_3982+1242::*gfp* | | | This study |  |
| Xoc_3982*::gfp | *Xoc*_3982+1242::*gfp* ;*xoc_3982* with ACG to GTT mutation | | | This study |  |
| ΔXonc3711-X+3 | sRNA Xonc3711 deletion mutant containing *xoc_3982*+3::*gfp* | | | This study |  |
| ΔXonc3711-X+1242 | sRNA Xonc3711 deletion mutant containing *xoc_3982*+1242::*gfp* | | | This study |  |
| Xonc3711^OE^ | Xonc3711 overexpressing strain, contains pHM1-Xonc3711, Sp^R^ | | | This study |  |
| ΔXonc3711-pXonc | sRNA Xonc3711 deletion mutant containing pHM1-Xonc3711, Sp^R^ | | | This study |  |
| Δ3982-pXonc | *xoc_3982* deletion mutant harboring pHM1-Xonc3711, Sp^R^ | | | This study |  |
| Xonc3711^＊^ | Xonc3711 with UGC to CAA mutation | | | This study |  |
| 3982^＊^ | *xoc_3982* with ACG to GTT mutation | | | This study |  |
| ΔXonc3711-3982^＊^ | sRNA Xonc3711 deletion mutant containing *xoc_3982* with ACG to GTT mutation | | | This study |  |
| Xonc3711^＊^-3982^＊^ | Contains Xonc3711 with UGC to CAA mutation and *xoc_3982* with ACG to GTT mutation | | | This study |  |
| ΔRNaseEC | RNase E deletion mutant lacking the C-terminal scaffolding region | | | This study |  |
| ΔRNaseECΔXonc3711 | Double mutant containing deletion in C-terminal scaffolding region of RNase and a deletion in Xonc3711 | | | This study |  |
| ΔXopC2 | *xopC2* deletion mutant of BLS256 | | | This study |  |
| C-ΔXopC2 | Complemented ΔXopC2 mutant; contains *xopC2* in trans | | | This study |  |
| ΔfliC | *fliC* deletion mutant of BLS256 | | | This lab |  |
| xopC2* | xopC2 promoter mutant, xopC2 with GCTTT to ATCGC mutation of BLS256 | | | This study |  |
| Plasmids | | | | |  |
| pKMS1 | Km^R^; R6K-based suicide vector; requires the *pir*-encoded π protein for replication | | [2] | |  |
| pHM1 | Sp^R^, *Mob*(*p*)*, IncW, Mob*^+^*, LaclP*^+^, PK2 replicon, cosmid | | [3] | |  |
| pHM1-Xonc3711 | BLS256 Xonc3711 cloned in pHM1, Sp^R^ | | This study | |  |
| pHM1-XopC2 | BLS256 XopC2 cloned in pHM1, Sp^R^ | | This study | |  |
| pKMS1::ΔXonc3711 | Xonc3711 deletion cassette in pKMS1, Km^R^ | | This study | |  |
| pKMS1::X+3 | *xoc_3982* with a *gfp* mutation at the 3^th^ amino acid cloned in pKMS1, Km^R^ | | This study | |  |
| pKMS1::X+1242 | *xoc_3982* with a *gfp* mutation at the 1242^th^ amino acid cloned in pKMS1, Km^R^ | | This study | |  |
| pKMS1::Δ*hfq* | *hfq* deletion cassette in pKMS1, Km^R^ | | This study | |  |
| pKMS1::Δ3982 | *xoc_3982* deletion cassette in pKMS1, Km^R^ | | This study | |  |
| pKMS1::Xonc3711^＊^ | Xonc3711 with UGC to CAA mutation cloned in pKMS1,Km^R^ | | This study | |  |
| pKMS1::3982^＊^ | *xoc_3982* with ACG to GTT mutation cloned in pKMS1, Km^R^ | | This study | |  |
| pKMS1::ΔRNaseEC | BLS256 C-terminal scaffolding region of RNase E deletion cassette in pKMS1, Km^R^ | | This study | |  |
| pKMS1::xopC2* | xopC2 with GCTTT to ATCGC mutation cloned in pKMS1, Km^R^ | | This study | |  |
| pHM1::Xonc3711 | BLS256 Xonc3711 cloned in pHM1, Sp^R^ | | This study | |  |
| pET-30a | N-His, N-thrombin, N-T7, C-His, Km^r^ | | Lab stock | |  |
| pET-30a::*hfq* | BLS256 *hfq* cloned in pET-30a, Km^r^ | | This study | |  |
| pET-30a::3982 | BLS256 *Xoc_3982* cloned in pET-30a, Km^r^ | | This study | |  |
| pHM1::*gfp* | *gfp* cloned in pHM1, Sp^R^ | | Lab stock | |  |

**REFERENCES**

1. Bogdanove A, Koebnik R, Lu H, Furutani A, Angiuoli S, Patil P, et al. Two New Complete Genome Sequences Offer Insight into Host and Tissue Specificity of Plant Pathogenic *Xanthomonas* spp. J Bacteriol. 2011;193:5450-5464.

2. Li Y-R, Zou H, Che Y-Z, Cui Y-P, Guo W, Zou L-F, et al. A Novel Regulatory Role of HrpD6 in Regulating hrp-hrc-hpa Genes in *Xanthomonas oryzae* pv. *oryzicola*. MPMI. 2011;24:1086-1101.

3. Innes R, Hirose M, Kuempel P. Induction of nitrogen-fixing nodules on clover requires only 32 kilobase pairs of DNA from the *Rhizobium trifolii* symbiosis plasmid. J Bacteriol. 1988;170:3793-802.
