# Supplementary material for: A key antisense sRNA modulates the oxidative stress response and virulence in Xanthomonas oryzae pv. oryzicola
Source: PLoS Pathog. 2021 Jul 23;17(7):e1009762. doi: 10.1371/journal.ppat.1009762 (PMC8336823; doi:10.1371/journal.ppat.1009762)
Supplement: S2 Table — (DOCX) [file ppat.1009762.s006.docx]

**S2 Table.** Primers used in this study.

| **Primers** | **Sequence (from 5’ to 3’)^a^** | **Application** | **Source** |
| --- | --- | --- | --- |
| F-Xonc3711uh(Xb) | GCTCTAGAGCTTCCCACACAACCGCCGC | Upstream homologous arm of Xonc3711 | This study |
| R-Xonc3711uh | GCTGAATCGCAGAGCGCTTAGCGTAAGATT |  | This study |
|  |  |  |  |
| F-Xonc3711dh | TACGCTAAGCGCTCTGCGATTCAGCTAAAC | Downstream homologous arm of Xonc3711 | This study |
| R-Xonc3711dh(Sp) | CATGCATGCGACCTCGCCAAGGGGCATCC |  | This study |
|  |  |  |  |
| F-X3uh(Ba) | CGGGATCCTTGCGCGATGACGGCGTGCT | Upstream homologous arm of X+3 | This study |
| R-X3uh | GCTCACCATCACGCGGGCAGCATGCAGCGG |  | This study |
|  |  |  |  |
| F-X3*gfp* | TGCCCGCGTGATGGTGAGCAAGGGCGAGGA | *gfp* fragment | This study |
| R-X3*gfp* | TCGAGGATTTTCTTGTACAGCTCGTCCATG |  | This study |
|  |  |  |  |
| F-X3dh | CGAGATTTAAATGAAGACAGTGATCCGCA | Downstream homologous arm of X+3 | This study |
| R-X3dh(Sa) | ACGCGTCGACTCCAGAATGCTGCGGTCGTT |  | This study |
|  |  |  |  |
| F-X1242uh(Ba) | CGGGATCCTGCCTCGTCCAAATTGCC | Upstream homologous arm of X+1242 | This study |
| F-X1242uh | GCCCTTGCTCACGTCGAACAGGCTCGCCTG |  | This study |
|  |  |  |  |
| F-X1242*gfp* | AGCCTGTTCGACGTGAGCAAGGGCGAGGAG | *gfp* fragment | This study |
| R-X1242*gfp* | CATGGCAAGTCACTTGTACAGCTCGTCCAT |  | This study |
|  |  |  |  |
| F-X1242dh | GAGCTGTACAAGTGACTTGCCATGTTCAGC | Downstream homologous arm of X+1242 | This study |
| R-X1242dh(Sa) | ACGCGTCGACATCCAGTGCTCGGGCTCG |  | This study |
|  |  |  |  |
| F-*hfq*uh(Ba) | CGCGGATCCGCAGGCGGCGGTCGGTTTCC | Upstream homologous arm of *hfq* | This study |
| R-*hfq*uh | GGACCGGCTGGGGCGGAGTAGTGCGTGTTT |  | This study |
|  |  |  |  |
| F-*hfq*dh | CGCACTACTCCGCCCCAGCCGGTCCCGGAA | Downstream homologous arm of *hfq* | This study |
| R-*hfq*dh(Sp) | ACATGCATGCTGGAGGTCTACCGCATCAGC |  | This study |
|  |  |  |  |
| F-3982uh(Ba) | CGCGGATCCCGGCGCTACCCGGTCCACGATC | Upstream homologous arm of *xoc_3982* | This study |
| R-3982uh | GCATGCTGCCCGCCTTGCCATGTTCAGCGC |  | This study |
|  |  |  |  |
| F-3982dh | AACATGGCAAGGCGGGCAGCATGCAGCGGTG | Downstream homologous arm of *xoc_3982* | This study |
| R-3982dh(Ps) | AAAACTGCAGGACTTTCGCCACGGCTACAG |  | This study |
|  |  |  |  |
| F-xopC2uh(Ba) | CGCGGATCCTTTCGCCACTACCAGTTTGC | Upstream homologous arm of *xopC2* | This study |
| R-xopC2uh | CTGGAGCACGGTAGACCGCAGCAAGGAGTAGTGC |  | This study |
|  |  |  |  |
| F-xopC2dh | TTGCTGCGGTCTACCGTGCTCCAGTGGCTCGCGG | Downstream homologous arm of *xopC2* | This study |
| R-xopC2dh(Sa) | ACGCGTCGACAGTTGTTGTTGAAAGCGGGC |  | This study |
|  |  |  |  |
| F-Xonc3711^＊^ | GACGAATTCAAATAATTCAAACGTATCCTTCG | Xonc3711 point mutation | This study |
| R-Xonc3711^＊^ | TTGAATTATTTGAATTCGTCGGCGAGAGAGAG |  | This study |
|  |  |  |  |
| F-3982^＊^ | GGAGGCACAGGAAGCGTTTGATTTGGCGGCGT | *xoc_3982* point mutation | This study |
| R-3982^＊^ | CAAACGCTTCCTGTGCCTCCAGCGGTGCCAAC |  | This study |
|  |  |  |  |
| F-xopC2 | GCGGCCGAAACCCCGGTGCGTACCGCGACC | *xopC2* promoter point mutation | This study |
| R-xopC2 | TACGCACCGGGGTTTCGGCCGCCAGCGCGGC |  | This study |
|  |  |  |  |
| F-RNaseECuh(Ba) | CGGGATCCAAGGCTGACACCGACCACGA | Upstream homologous arm of C-terminal scaffolding region of RNaseE | This study |
| R-RNaseECuh | ACCAGCTCATCAGCGCTGCTGCCGGTCAGCGT |  | This study |
|  |  |  |  |
| F-RNaseECdh | CGGCAGCAGCGCTGATGAGCTGGTGATCGACC | Downstream homologous arm of C-terminal scaffolding region of RNaseE | This study |
| R-RNaseECdh(Ps) | AAAACTGCAGTCAGTGGCGTCAGTTGGATT |  | This study |
|  |  |  |  |
| F-pHM1(Ec) | CGGAATTCTAAAAAAATCGCGCCAGA | Overexpression of Xonc3711 | This study |
| R-pHM1(H) | CCCAAGCTTCGTTGCTTGCTATGACAG |  | This study |
|  |  |  |  |
| F-*hfq*(Ba) | CGCGGATCCATGGCTAAGGGGCAATCT | Hfq purification | This study |
| R-*hfq*(Xh) | CCGCTCGAGCTGCTCGACGTCGTCATC |  | This study |
|  |  |  |  |
| F-3982(Ba) | CGGGATCCGTGAAAATCCTCGACAAGCT | Xoc_3982 protein purification | This study |
| R-3982(Xh) | CCGCTCGAGGTCGAACAGGCTCGCCTGCC |  | This study |
|  |  |  |  |
| 5S rRNA | CTGGCAGGCTTAGCTTCCGGGTTCGGGATG | Digoxigenin-labeled DNA probes for Northern blot analysis | This study |
|  |  |  |  |
| Xonc3711/ Xonc3711* | CTTGCTATGACAGGCGGCAGCGTCAGGCTC |  | This study |
|  |  |  |  |
| Xoc_3982 | AACTCCGACTACACCATGGAGCAGCTGG |  | This study |
|  |  |  |  |
| F-T7Xoc_3982 | TAATACGACTCACTATAGGGGTGAAAATCCTCGACAAG | Transcription of *Xoc_3982* and labeling T7 promoter for EMSA | This study |
| R-T7Xoc_3982 | GATTACAAGGATGACGACGATAAGCCGGGATGGTCTTGAGGT |  | This study |
|  |  |  |  |
| F-T7Xonc3711 | TAATACGACTCACTATAGGATTTTTTTAGCGCGGTC | Transcription of Xonc3711 and labeling T7 promoter for EMSA | This study |
| R-T7Xonc3711 | GCAACGAACGATACTGTCC |  | This study |
|  |  |  |  |
| *rpoD* F | CGACAACACCACCAACATCAATC | qPCR analysis of *rpoD* | This study |
| *rpoD* R | GCTTACCGACCTCTTCCAACG |  | This study |
|  |  |  |  |
| *FlgA* F | CCTGCTCGTCATCCTGTTGG | qPCR analysis of *flgA* | This study |
| *FlgA R* | GAGCGTGGCTTCGGCTTCAG |  | This study |
|  |  |  |  |
| *FliF* F | CCCATCCCTTTACGACTTGT | qPCR analysis of *fliF* | This study |
| *FliF R* | CGCCAGCATCGGACAGGAGC |  | This study |
|  |  |  |  |
| *FliM* F | GAGTCCTGGGAGGGAACGGC | qPCR analysis of *fliM* | This study |
| *FliM R* | AGGTGACCCTGTCCAGCGTG |  | This study |
|  |  |  |  |
| *FlhA* F | ACCAGCGCTTCCACAATCTT | qPCR analysis of *flhA* | This study |
| *FlhA R* | GGTCGCCACCCACCTGTCGC |  | This study |
|  |  |  |  |
| *FlhB* F | ATCCACTTCCAGCGAGGGCA | qPCR analysis of *flhB* | This study |
| *FlhB R* | CGTTGGCACGCGCCTTGTAT |  | This study |
|  |  |  |  |
| *FliC* F | CGGTGGTCTTGGCGTAGTCG | qPCR analysis of *fliC* | This study |
| *FliC R* | TTGGGCAGCCTGGATATCTC |  | This study |
|  |  |  |  |
| Xonc3711 F | ATTTTTTTAGCGCGGTC | qPCR analysis of Xonc3711 | This study |
| Xonc3711 R | GCAACGAACGATACTGTCC |  | This study |
|  |  |  |  |
| *xoc_3982* F | CTTCCACGCTGAACTCGTAG | qPCR analysis of *xoc_3982* | This study |
| *xoc_3982* R | CTGGACACCAGCCACAATCT |  | This study |
|  |  |  |  |
| *xopC2* F | CCTTTCGCGTAACGACTGCT | qPCR analysis of *xopC2* | This study |
| *xopC2* R | AATTCAAGGGAGCCGCTTCG |  | This study |

^a^ Underscored sequences indicate introduced restriction sites. Ba, *Bam*HI; Ec, *Eco*RI; H, *Hind*III; Ps, *Pst*I; Sa, *Sal*I; Sp, *Sph*I; Xb, *Xba*I; and Xh, *Xho*I. The nucleotides in red font indicate sites where point mutations were introduced.
